# Kinematic Variables Evolution During a 200-m Maximum Test in Young Paddlers

**DOI:** 10.2478/hukin-2013-0041

**Published:** 2013-10-08

**Authors:** Raquel Vaquero-Cristóbal, Fernando Alacid, Daniel López-Plaza, José María Muyor, Pedro A. López-Miñarro

**Affiliations:** 1 Chair of Sport Traumatology. Catholic University of San Antonio of Murcia. Spain.; 2 Department of Physical Activity and Sports. Catholic University of San Antonio of Murcia. Spain.; 3 Department of Sport, Coaching and Exercise Science. University of Lincoln. England.; 4 Laboratory of Kinesiology, Biomechanics and Ergonomics (KIBIOMER). University of Almería. Spain.; 5 Department of Physical Education. University of Murcia. Spain.

**Keywords:** Speed evolution, cycle frequency, cycle length, cycle index, kayakers, canoeists

## Abstract

The objective of this research was to determine the kinematic variables evolution in a sprint canoeing maximal test over 200 m, comparing women and men kayak paddlers and men canoeists. Speed evolution, cycle frequency, cycle length and cycle index were analysed each 50 m section in fifty-two young paddlers (20 male kayakers, 17 female kayakers and 15 male canoeists; 13–14 years-old). Recordings were taken from a boat which followed each paddler trial in order to measure the variables cited above. Kinematic evolution was similar in the three categories, the speed and cycle index decreased through the test after the first 50 m. Significant differences were observed among most of the sections in speed and the cycle index (p<0.05 and <0.01, respectively). Cycle length remained stable showing the lowest values in the first section when compared with the others (p<0.01). Cycle frequency progressively decreased along the distance. Significant differences were identified in the majority of the sections (p<0.01). Men kayakers attained higher values in all the variables than women kayakers and men canoeists, but only such variables as speed, cycle length and cycle index were observed to be significantly higher (p<0.01). Moreover, lower kinematic values were obtained from men canoeists. The study of the evolution of kinematic variables can provide valuable information for athletes and coaches while planning training sessions and competitions.

## Introduction

In recent years there has been an increasing interest in the quantitative analysis of technique in cyclical sports which has led to a better understanding of the evolution of velocity. The aim of these studies was to determine the optimal relationship between fatigue in athletes and their performance (Craig et al., 1985). Following this trend, Brown et al. (2010) investigated the influence of performance level and exercise mode on the rowers’ pacing strategies during a 2000 m maximum test. They found that on water, intermediate placements highlighted the significant influence of taking the front of the race (at the elite level 78% of the winners were first at the middle of the race and 100% were in the top three). First investigations about quantitative analysis came originally from competitive swimming. Much attention has been paid to the interrelationships of mean velocity, stroke rate and stroke length in swimming (Craig and Pendergast, 1979; Keskinen et al., 1989). Subsequently, Costill et al. (1985) introduced the concept of the cycle index (stroke length by velocity), as an index of efficiency and economy of the cycle. Researchers have reported that swimmers adjust the majority of race variables such as average swim velocity, stroke frequency, and stroke length to race distance (Cappaert, 1999; Keskinen and Komi, 1993; Pelayo et al., 1996). After that, quantitative analyses were performed in other sports such as cycling (Hettinga et al., 2009). Recently, researchers have shown an increasing interest in sprint kayaking. Investigations have taken into account quantitative aspects (Alacid et al., 2005). Thus, first studies of kayakers investigated the evolution of different kinematic variables separately, analyzing the boat velocity over different distances (Alacid and Carrasco, 2004; Bishop et al., 2002; Issurin, 1998), the cycle frequency on kayakergometer and on open water (Alacid et al., 2006; Barnes and Adams, 1998; Van Someren and Oliver, 2002), and the differences between the “wash riding” technique and paddling alone (Gray et al., 1995; Pérez-Landaluce et al., 1998). Later studies have focused on the relationship between the evolution of several of these kinematic variables and their influence on each other. Sealey et al. (2011) observed the effect of self-selected and induced slow and fast paddling on stroke kinematics during 1000 m outrigger canoeing ergometry. Stroke length and stroke time were negatively correlated to the stroke rate.

The study of the evolution of boat velocity, stroke rate, stroke length and split times provided some important information to athletes and coaches (Sperlich and Baker, 2002). To collect kinematic data in situ, various methods have been proposed. Sperlich and Baker (2002) indicated that valuable information could be obtained from video recordings, which are taken from a boat or vehicle following the race on one side and parallel to the paddlers. With a time overlay and using the buoys on the course as a distance measure, kinematic race data can be calculated. Race analysis data can be used not only during competition to provide feedback to the athlete with regard to their performance and possible strategies for races in a regatta, but also to plan future races. The success of the German Sprint Canoe team at the 1992 Barcelona Olympics Games can be in part attributed to the scientific support during the regatta and also in the buildup to the Games (Sperlich and Baker, 2002).

Therefore, the beneficial effect of kinematic analysis of the results in the competition has been proved. However, research has focused on elite athletes, with fewer studies focusing on young paddlers. It is important to know the evolution of these variables in young paddlers and to compare these with the results obtained by professional athletes. The objective of this research was to determine the kinematic evolution of velocity, cycle frequency, cycle length and cycle index in a sprint canoeing maximal test over 200 m, comparing women and men kayak paddlers and men canoeists.

## Material and Methods

### Participants

Fifty-two young paddlers (20 male kayakers, 17 female kayakers and 15 male canoeists; 13–14 years-old) participated in this study. They were selected by the Royal Spanish Canoeing Federation as the best in their categories to participate in the 2008 National Development Camp. The Institutional Ethical Committee of the University of Murcia approved the study and written informed consent form was obtained from the parents of all the children before commencement of the study. The paddlers that took part in this research neither were presenting any disease nor were submitted to pharmacological treatment in the period in which the tests were carried out.

### Procedures

All paddlers performed a 200 m maximum test along a lane annotated with buoys every 25 m and positioned on both sides of the paddler’s movement. This allowed to divide the two hundred meters distance covered by the paddlers, into 50 m sections in order to analyze the evolution of boat velocity, cycle frequency, cycle length and cycle index along the races. Every participant used his/her habitual boat and paddle of training and competition. The instructions to determine the start of the test were the habitual indications used in canoeing. Before the beginning of the test, the paddle was already in the water. The paddler first traction movement was selected as the first frame to be analysed. So, reaction time was not taken into account. Each test was recorded using a JVC Everio MG-135 (Victor Company of Japan) video camera at 30 frames per second. The recordings were taken from a motorboat following the races from a lateral perspective and with one lane (5 m) of separation between the paddler and the motorboat when the camera was placed. After that, to analyse the data collected the VirtualDub 1.8.8 software by Avery Lee was used.

To obtain boat velocity information, the frame in which the bow of the kayak/canoe was aligned with the buoys was selected. These buoys indicated the beginning and the end of the sections analyzed. Then, the difference between the initial and final frame of each stage was calculated. The time taken to cover the section was calculated by dividing this number by 30. Finally, boat velocity (m/s) was obtained dividing 50 m by the time taken to cover the section. Cycle frequency (cycles/s) was calculated by counting the complete cycles performed in the section. In this case, the frames when the blade tip first contacted the water were selected. These frames were always higher or equal to the number of frames used to calculate the velocity. After that, the difference between the initial and the final frame was divided by 30. Then, the number of complete cycles was divided by this number. There were two additional circumstances taken into account in this analysis: 1) the first cycle of each race was excluded because the boat started from a static position, so this cycle was different to the rest; 2) to calculate the last cycle frequency, the frame when the blade tip last contacted the water surface before the finish line was used. The cycle length (m/cycle) of each section was calculated by dividing the kayak velocity between the cycle frequency obtained in a certain section. To calculate the cycle index (m^2^/(cycles·s)) the boat velocity was multiplied by the cycle length obtained in each section.

### Analyses

Data were analysed using the Statistical Package for Social Sciences (SPSS Inc, version 15.0, Chicago, ILL, USA) via repeated measures ANOVA and post hoc Bonferroni tests. Reliability and within-subject variability for mean and peak responses were assessed using intraclass correlation coefficients (ICC) and measures bias/ratio with associated 95% limits of agreement (LOA), respectively. Data are presented as mean ± SD with alpha set at 0.05.

## Results

Boat velocity values for the four sections are shown in Table 1. What is worth mentioning is that boat velocity evolution was similar for all paddlers with a first section slower and a second section faster than the others. After that, the velocity decreased significantly (p<0.05) in the two final sections, except in the last section of the men canoe test. There were significant differences (p<0.05) in men and women kayakers in the first section when compared with the others and among all groups comparing the second section with the 100 to 150 m section. Comparisons between the groups demonstrated significant differences in velocity (p<0.01) because men kayakers were the fastest in all sections. In addition, significant differences (p<0.01) were found between women kayakers and men canoeists in the 50 to 100 m and in the 100 to 150 m sections. There were also significant differences between them in the 150 to 200 m section (p<0.05). Conversely, no significant differences were found in the first section.

The cycle frequency shown in Table 2 decreased along the test in all paddlers, finding the highest values in the first section. There were significant differences (p<0.01) between the first and the two last sections in kayakers and among all other sections in canoeists. Additionally, significant differences (p<0.01) between the 50 to 100 m and 100 to 150 m, the 50 to 100 m and 150 to 200 m, and the 100 to 150 m and 150 to 200 m sections in men and women kayakers were found. Examining the evolution of cycle frequency among groups, no significant differences between men and women kayakers and between women kayakers and men canoeists were found. Between men kayakers and men canoeists there were only significant differences (p<0.05) in the second section.

Cycle length values for the four sections are shown in Table 3. The evolution of this variable was characterized by maintaining its stability from the second section with the highest values in the first section (0 to 50 m). All groups showed significantly higher (p<0.01) values in this section when compared with others. Moreover, there were significant differences (p<0.01) between men kayakers and men canoeists in all sections and between men and women kayakers in the last two sections. Comparisons between women kayakers and men canoeists showed that there were significant differences in the 0 to 50 m and in the 150 to 200 m section (p<0.05) and in the two intermediate sections (p<0.01).

Cycle index (m^2^/(cycles·s)) values for the four sections are presented in Table 3. The cycle index is the product of boat velocity and cycle length. Because of the fact that the second section showed a high stability during the test, the variable had a similar evolution to the velocity. In addition there were significant differences (p<0.01) between men and women kayakers, men kayakers and men canoeists in all sections and between women kayakers and men canoeists in the 50 to 100 m and the 100 to 150 m sections. In the first and the last sections the difference was less than in the intermediate sections (p<0.05).

## Discussion

This study has shown that variables evolution was similar in all studied groups (men and women kayakers and men canoeists). The results of this study with regard to the velocity indicated that the first section was the slowest during the test. This may be due to the influence of the start in the 0 to 50 m section. It was found that the second section was the fastest in all groups. After that, the velocity decreased progressively, except in the last section of the men canoe test. In reviewing previous research, similar velocity evolution was found in international paddlers in a 1000 m test (Alacid and Carrasco, 2004; Sánchez and Magaz, 1993) and in rowers during a 2000 m maximum test (Garland, 2005). The parabolic-shaped evolution depicted by the kayakers velocity was similarly found in other research undertaken with rowers (Muehlbauer and Melges, 2011; Muehlbauer et al., 2010). Muehlbauer and Melges (2011) indicated that the absence of a final spurt suggests that the physiological status of the athlete plays an important role in controlling the timing and rate of the decline in rowing speed. Additionally, Muehlbauer et al. (2010) defined that the parabolic-shaped race profile indicates an anticipatory control of speed and energy distribution over the course of the race. The observed changes in pacing pattern suggest that during the finals a more conservative starting pace is used, which could be physiologically advantageous, because some energy is reserved for the final spurt. Further research by Hanon and Thomas (2011) analyzed strategies adopted by elite runners during 400, 800 and 1500 m tests to optimize performance. They found that final velocity decreased significantly for all three distances similarly to the kayakers in the current study. This decrease was correlated with diminished oxygen uptake (VO2), the reduction in tidal volume and blood lactate concentrations. In addition, there are similarities between the velocity evolution values in canoeists and those described by Noakes et al. (2009). They analysed the pacing strategies adopted by world-record breakers during a one mile footrace finding that mean times for the second and third laps were both slower than those for the first or final laps. The fact that the final lap was found as the fastest one, similarly to the canoeists in the present study, leads us to suspect that pacing strategies are regulated “in anticipation” and are not purely the result of a developing “peripheral fatigue”. Micklewright et al. (2010) indicated that pacing is influenced by an interaction between feedback and previous experience. Conscious cognitive processes that lead to high ratings of perceived exertion and pacing appear to be influenced by previous experience.

Cycle frequency decreased along the test in all paddlers. In our study there were no significant differences between groups in most cases. Very little was found in the literature regarding cycle frequency evolution. Issurin (1998) investigated competitive performance patterns for 228 world best kayakers who took part in the World Championships from 1983 to 1997, and in the Olympic Games of Seul (1988) and Atlanta (1996). Four different models of evolution of cycle frequency were determined. Surprisingly, cycle frequency evolution values obtained from our study were similar to those obtained by Issurin (1998). Sealey et al. (2011), according to Craig and Pendergast (1979), found that cycle length and cycle time were negatively correlated to cycle frequency. The analysis of cycle length and cycle frequency at constant velocity demonstrated an increase in cycle frequency whereas cycle length slightly decreased along the distance as a consequence of the fatigue of the swimmer (Craig and Pendergast, 1979; Craig et al., 1985; Guimaraes and Grimston, 1983). Also Keskinen et al. (1989) encountered in short maximum swims that cycle length is a determinant factor in producing very high subsequent swimming velocity. In agreement with prior research in swimming, the evolution of cycle length was characterized by maintaining its stability or slightly decreasing along the race except for the first section which showed the lowest values. This may be due to the influence of the start in the 0 to 50 m section as in the start the paddlers use shorter strokes to achieve greater acceleration. In a 500 m maximum test in kayakers, Alacid et al. (2005) found a similar cycle length evolution, characterized by a first section with shorter strokes and a gradual decrease of the variable from the second section.

As the cycle index is defined as the product of boat velocity and cycle length, this variable resulted in a similar evolution to boat velocity due to the stability of cycle length from the second section. Similar results were found in a 500 m maximum test in kayakers by Alacid et al. (2005).

To sum up, the most interesting finding of this paper was the identification of kinematic variable evolution in young paddlers. Some authors have speculated about the need to obtain a kinematic pattern to optimize the relationship between the evolution of kinematic variables (velocity, cycle frequency, cycle length and cycle index) and fatigue in athletes and their performance (Craig et al., 1985). Previous studies had studied the evolution of these variables separately over different distances (Alacid and Carrasco, 2004; Alacid et al., 2006; Barnes and Adams, 1998; Bishop et al., 2002; Issurin, 1998; Van Someren and Oliver, 2002). The present study was designed to determine the relationship between kinematic variables in a maximum test.

Furthermore, coaches might use the proposed method to easily collect kinematic data with high reliability and validity. Nowadays, trainers obtain kinematic variables manually using low reliable stopwatches and stroke counters (Alacid et al., 2011). The kinematic variables obtained can be useful to train with an ideal relationship between cycle frequency and length; to establish race strategies; to determine when the onset of fatigue affects paddler’s performance and decreases their velocity; to monitor the progress of the paddler; to optimize kayak equipment; and to configure team boats (Alacid et al., 2011). Additionally, the recording might be used to conduct a qualitative analysis of padding technique (Alacid et al., 2011).

## Conclusion

The study of the evolution of kinematic variables can provide athletes and coaches with some valuable information on how to plan the training sessions and competitions looking for the optimal use of their resources. The foregoing discussion has attempted to assess kinematic variables evolution during a 200 m maximum test. This study has shown that the variables evolution was similar in all groups (men and women kayakers and men canoeists). A decreasing tendency in the velocity and the cycle index after the first 50 m was found. In both variables significant differences were observed among most of the sections. It was also shown that cycle frequency decreased along the test (significant differences among most of the sections were found) and that cycle length was stable, except for the first section where values were significantly the lowest along the test. Men kayaker values were significantly higher than those obtained by other paddlers, while men canoeists showed the lowest values in speed, cycle length and cycle frequency, followed by women kayakers. No differences between groups were found in all kinematic variables except for cycle frequency.

## Figures and Tables

**Table 1:** Boat velocity (m/s) mean ± SD

Sections	Kayak men	Kayak women	Canoe men
0–50 m	3.60 ± 0.07	3.27 ± 0.05	3.06 ± 0.07
50–100 m	4.30 ± 0.07^*^	3.82 ± 0.05^*^	3.30 ± 0.07
100–150 m	4.04 ± 0.7^*†^	3.59 ± 0.05^*†^	3.09 ± 0.08^†^
150–200 m	3.92 ± 0.8^†‡^	3.48 ± 0.06^*‡^	3.15 ± 0.08

* p<0.05 with respect to the 0–50 m section;

† p<0.05 with respect to the 50–100 m section;

‡ p<0.05 with respect to the 100–150 m section.

**Table 2:** Cycle frequency (cycles/s) mean ± SD

Sections	Kayak men	Kayak women	Canoe men
0–50 m	1.05 ± 0.01	1.01 ± 0.01	1.06 ± 0.30
50–100 m	1.02 ± 0.01	0.98 ± 0.01	0.94 ± 0.20^*^
100–150 m	0.96 ± 0.01^*†^	0.94 ± 0.01^*†^	0.90 ± 0.20^*^
150–200 m	0.91 ± 0.01^*†‡^	0.90 ± 0.01^*†‡^	0.90 ± 0.20^*^

* p<0.01 with respect to the 0–50 m section;

† p<0.01 with respect to the 50–100 m section;

‡ p<0.01 with respect to the 100–150 m section.

**Table 3:** Cycle length (m/cycle) mean ± SD and Cycle index (m^2^/(cycles·s)) mean ± SD

Sections	Kayak men	Kayak women	Canoe men
0–50 m Cycle length	3.41 ± 0.07	3.24 ± 0.06	2.91 ± 0.09
50–100 m Cycle length	4.20 ± 0.06^*^	3.90 ± 0.07^*^	3.51 ± 0.10^*^
100–150 m Cycle length	4.20 ± 0.06^*^	3.81 ± 0.07^*^	3.44 ± 0.09^*^
150–200 m Cycle length	4.29 ± 0.08^*^	3.87 ± 0.07^*^	3.55 ± 0.12^*^
0–50 m Cycle index	12.39 ± 0.42	10.67 ± 0.35	8.94 ± 0.41
50–100 m Cycle index	18.17 ± 0.49^*^	14.99 ± 0.42^*^	11.63 ± 0.52^*^
100–150 m Cycle index	17.06 ± 0.51^*‡^	13.74 ± 0.45^*†^	10.72 ± 0.49^*‡^
150–200 m Cycle index	16.92 ± 0.59^*†^	13.55 ± 0.46^*†^	11.23 ± 0.52^*^

* p<0.01 with respect to the 0–50 m section;

† p<0.01 with respect to the 50–100 m section;

‡ p<0.05 with respect to the 50–100 m section.
